# Supplementary material for: EPIC-TRACE: predicting TCR binding to unseen epitopes using attention and contextualized embeddings
Source: Bioinformatics. 2023 Dec 9;39(12):btad743. doi: 10.1093/bioinformatics/btad743 (PMC10963061; doi:10.1093/bioinformatics/btad743)
Supplement: btad743_Supplementary_Data [file btad743_supplementary_data.pdf]

Supplementary material to EPIC-TRACE: predicting TCR binding to unseen epitopes using attention and contextualized embeddings

Dani Korpela[1,*], Emmi Jokinen[1,2,3], Alexandru Dumitrescu[1], Jani Huuhtanen[2,3], Satu Mustjoki[2,3,4], and Harri Lähdesmäki[1,*]

[1]Department of Computer Science, Aalto University, Espoo, 02150, Finland.
[2]Translational Immunology Research program and Department of Clinical Chemistry and Hematology,, University of Helsinki, Helsinki, 00290, Finland
[3]Hematology Research Unit Helsinki, Helsinki University Hospital Comprehensive Cancer Center, Helsinki, 00290, Finland
[4]iCAN Digital Precision Cancer Medicine Flagship, Helsinki, Finland
[*]Correspondence

S1 TCRconv comparison

In the first setting we trained and evaluated TCRconv with the 30 least frequent epitopes that had at least 27 TCRs (TCRconv27) or 45 TCRs (TCRconv45) in the train set and with the 30 most frequent epitopes (TCRconv30mf) (See Epi27, Epi45 and Epi780, respectively Table S1). In this setting TCRconv was trained utilizing only the β chain as all datapoints did not contain α chain information. TCRconv used the full contextualised chain (similarly as EPIC-TRACE) when available. EPIC-TRACE was trained on the full $\mathcal{D}_{\alpha\beta,\beta}$ cross-validation train sets but tested only on the 30 corresponding epitopes such that the testing data was identical. This was done both using all available features and comparing using a reduced model with only the β chain and without the MHC information.

In the second setting the cross-validation was stratified such that the train set contained always at least 45 (positive) TCRs with both chains available for each epitope. Here both methods were trained utilizing both chains and using the full context if available (precise V and J genes). To show the benefit of using single chain datapoints in addition to the $\alpha\beta$ datapoints we trained EPIC-TRACE also by adding the α and β datapoints corresponding to the epitopes already in the train set. Lastly we added all other datapoints to the train set of EPIC-TRACE. From Table S3 we can see that when training on the same data TCRconv performs better on both AUROC and AP (rows 1 and 4) . However, as corresponding single chain datapoints are added to the train set, both scores are improved and EPIC-TRACE performs better in terms of AUROC (row 2). Further adding other epitopes does not increase the performance on the epitopes

in test (row 3), which could be due to the already sufficient amount of data for the specific epitopes in the test set.

S2 Model specifics

First 1D convolutional layers (α, β and Epitope) have 100 output channels a kernel size of 7 and stride 1. The convolutional layers are followed by Dropout with a rate of 0.2. The Multi-head self attention modules (α-Epitope and β-Epitope) have 5 heads and use a dropout rate of 0.2. All allele information ($\beta_{\mathrm{V}}, \beta_{\mathrm{J}}, \alpha_{\mathrm{V}}, \alpha_{\mathrm{J}}$ and MHC) is embedded with the linear layers to dimension 8. The following linear layers after concatenation (α and β branches) have dimensions 2924×54 and a dropout rate of 0.45. These are followed by the output heads where the $\alpha\beta$ output head first has linear layer of dimension 108×36 with dropout rate 0.45 before the final linear layer and the sigmoid activation.

S3 Input embedding ablation

To investigate importance of the input embeddings we conducted an ablation study comparing one-hot, ProtBERT and concatenated ProtBERT-one-hot embeddings. The ProtBERT embeddings used in this ablation are utilizing the full (long) TCR context (when VJ are available). The results can be seen in Table S6.

S4 Figures and Tables

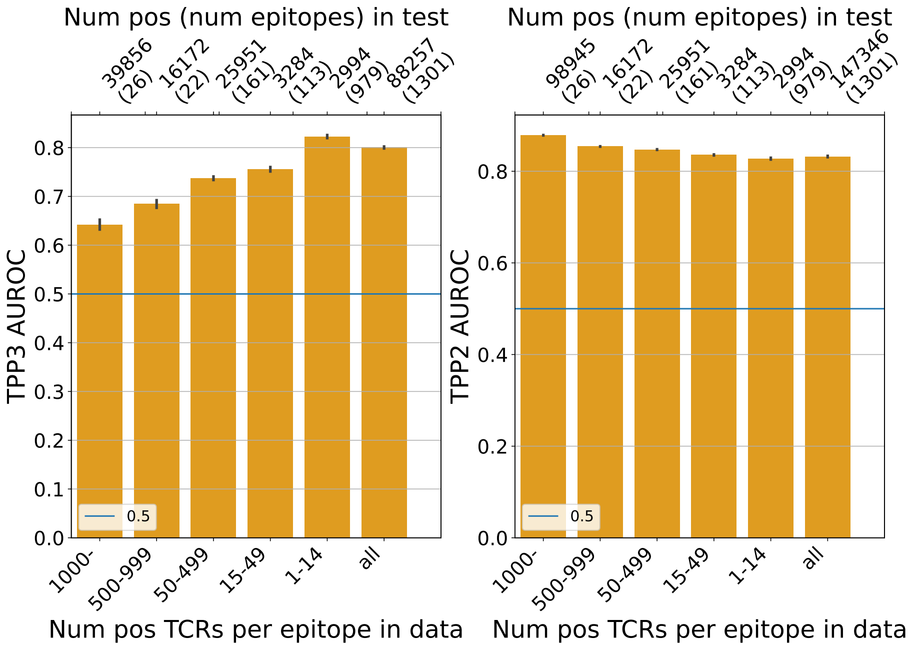

Figure S1: Average of per epitope AUROC values for epitopes binned by datapoint frequency in the TPP2 and TPP3 tasks. Epitopes were binned to five bins according to the number of positive datapoints to assess frequency based trend.

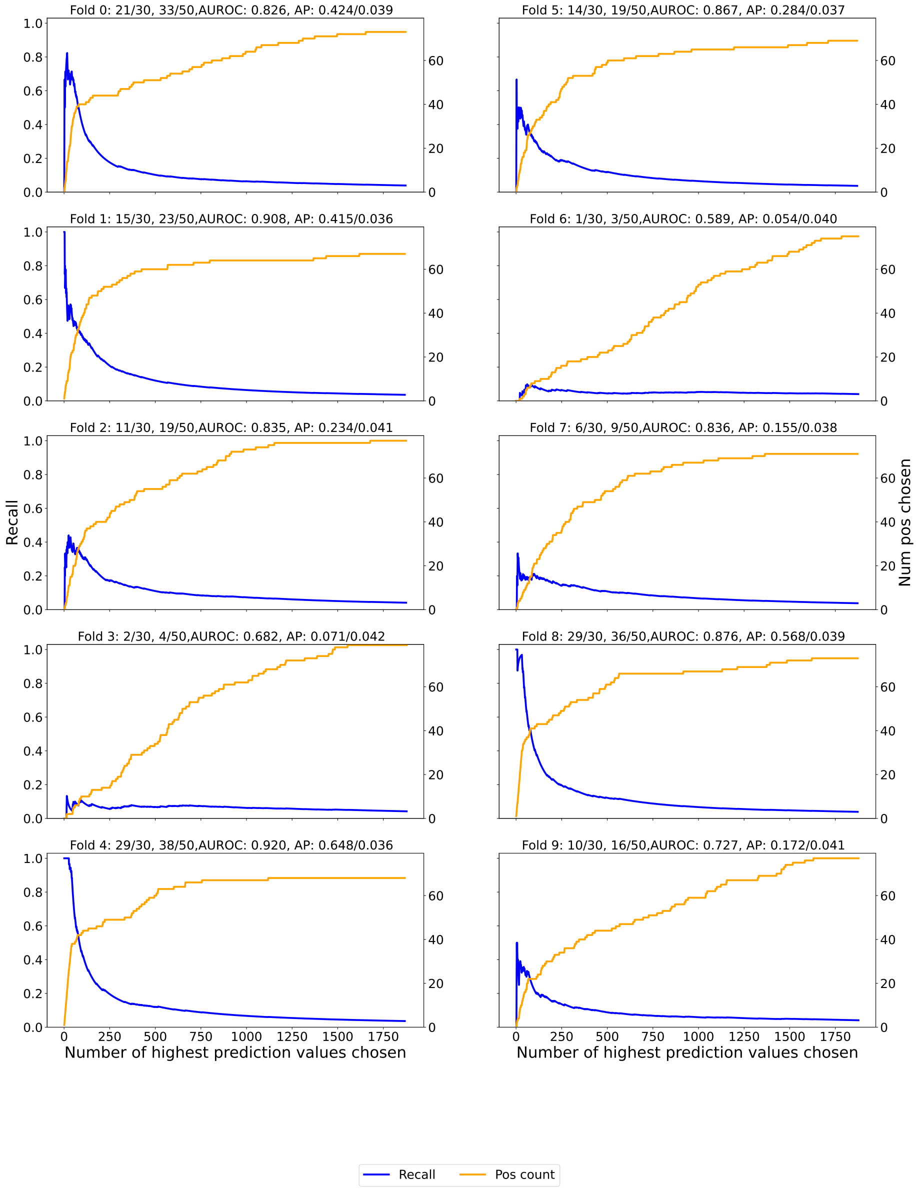

Figure S2: Recall and cumulative positive count by the number of highest chosen prediction values of all 10 parts of the yeast display experiment. The number of positives of the 30 and 50 highest prediction values are shown for each plot together with the AUROC and AP/RND_AP for each part.

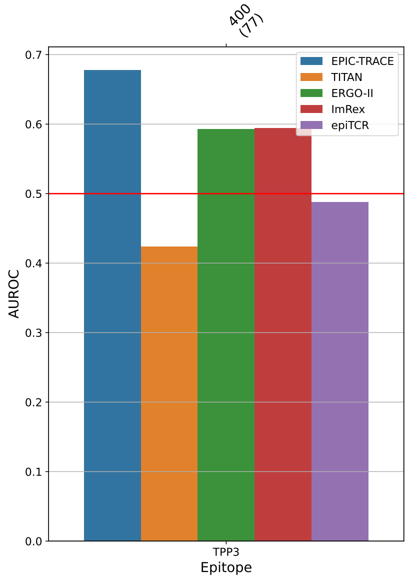

Figure S3: Results of independent test data. All datapoints added to IEDB and VDJDB after extraction of the base dataset $\mathcal{D}_{\alpha\beta,\alpha,\beta}$ was downloaded and filtered by requiring distinct epitope and βCDR3 sequence (TPP3)

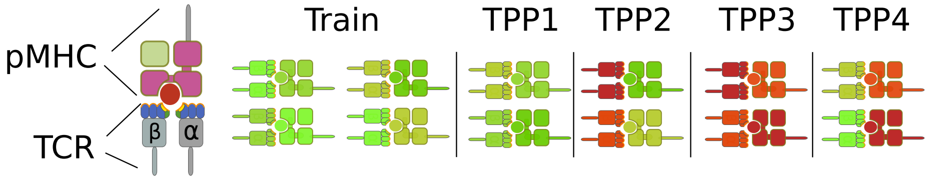

Figure S4: Illustration of TPP tasks, green TCRs and pMHCs are in train and red TCRs and pMHCs are distinct from those in train

Table S1: Dataset description. Number of unique features for the five IEDB + VDJdb based datasets and the external yeast display dataset ($\mathcal{D}_{\text{YD}}$).

	datapoints	β_{CDR3}	β_V	β_J	β_{long}	α_{CDR3}	α_V	α_J	α_{Long}	MHC	Epitope
$\mathcal{D}_{\alpha\beta,\beta}$	147346	118896	110	17	77022	19744	98	65	19765	69	1301
$\mathcal{D}_{\alpha\beta,\alpha,\beta}$	169792	118907	110	17	77022	34692	106	65	28390	69	1307
$\mathcal{D}_{\alpha\beta}$	28377	21266	106	17	21110	19744	98	65	19765	54	919
$\mathcal{D}_{\alpha\beta\geq 50}$	25178	18817	104	17	18727	17532	91	65	17606	15	32
Epi27	1181	1088	86	15	533	247	65	52	201	9	30
Epi45	1920	1786	85	15	1110	270	59	55	260	13	30
Epi780	102739	80538	102	17	58043	15629	84	64	15710	9	30
$\mathcal{D}_{\text{YD}}$	81	4	1	2	4	5	1	5	5	1	26

Table S2: Negative similarity and method comparison. All models were evaluated on two datasets were *(i)* negatives were generated from positives such that any change in the TCR (V, J or CDR3 or either chain) from a positive pair was defined as a plausible negative (TCR-similarity), and *(ii)* at least a difference in the βCDR3 region was required to generate a negative from the positives (β_{CDR3}-Similarity). TITAN an ImRex are only evaluated on the first of the five cross-validations. The difference between the negative generation methods is small and $< 1\%$ of the negatives have a corresponding positive βCDR3-Epitope pair.

		TPP2 AUROC	TPP2 AP	TPP3 AUROC	TPP3 AP
TCR-Similarity	$\alpha\beta$ (CDR3) + VJ +MHC	0.897 ± 0.000	0.676 ± 0.000	0.692 ± 0.007	0.289 ± 0.006
	ERGO-II	0.895 ± 0.002	0.659 ± 0.007	0.675 ± 0.007	0.274 ± 0.004
	TITAN	0.454	0.786	0.204	0.577
	ImRex	0.420	0.697	0.178	0.519
	epiTCR	0.793 ± 0.000	0.581 ± 0.000	0.515 ± 0.001	0.183 ± 0.001
β_{CDR3}-Similarity	$\alpha\beta$ (CDR3) + VJ +MHC	0.896 ± 0.000	0.678 ± 0.001	0.700 ± 0.008	0.294 ± 0.007
	ERGO-II	0.894 ± 0.002	0.653 ± 0.007	0.687 ± 0.007	0.278 ± 0.005
	TITAN	0.453	0.786	0.200	0.566
	ImRex	0.423	0.699	0.178	0.522
	epiTCR	0.792 ± 0.000	0.580 ± 0.000	0.516 ± 0.001	0.184 ± 0.000

Table S3: $\alpha\beta$ data ablation study. EPIC-TRACE was trained and tested on epitopes with at least 50 $\alpha\beta$ TCRs (32 epitopes). This was compared to a setting where single chain datapoints for the corresponding epitopes were added to the train set (row 2) and a setting where full data including other epitopes were included in the train set (row 3).

	AUROC	AP
$\alpha\beta$	0.843 ± 0.003	0.641 ± 0.006
$\alpha\beta$ ∪ corresponding α and β	0.853 ± 0.002	0.668 ± 0.004
Full data	0.853 ± 0.002	0.668 ± 0.005
TCRconv	0.848 ± 0.002	0.676 ± 0.006

Table S4: Comparison of validation strategies. The model was trained with either randomly selected validation data or validation data that had a unseen epitope (TPP3 or TPP4) relation to the remaining train set. Validation data was used for early stopping and selecting model parameters before SWA. Reported values are the mean and standard error of five 10-fold cross-validation runs.

	TPP3 AUROC	TPP3 AP
Random validation	0.691 ± 0.008	0.291 ± 0.005
Unseen epitope validation	0.688 ± 0.010	0.285 ± 0.008

Table S5: Comparison of models trained with all data or by discarding low frequency epitopes (i.e., epitopes with less than 15 TCRs) from training. Reported values are the mean of five 10-fold cross-validation runs together with the standard error. NA indicates that the setting is not consistent with the TPP2 task definition.

Test data	Train data	TPP2 AUROC	TPP2 AP	TPP3 AUROC	TPP3 AP
All	All	0.906 ± 0.000	0.698 ± 0.000	0.691 ± 0.008	0.291 ± 0.005
	<15 Discarded	NA	NA	0.681 ± 0.009	0.280 ± 0.008
≥ 15	All	0.907 ± 0.000	0.701 ± 0.000	0.686 ± 0.008	0.285 ± 0.005
	<15 Discarded	0.907 ± 0.000	0.701 ± 0.001	0.680 ± 0.009	0.278 ± 0.006

Table S6: Comparison of input embedding methods.

Model	TPP2 AUROC	TPP2 AP	TPP3 AUROC	TPP3 AP
Default ProtBERT+OH	0.906 ± 0.000	0.698 ± 0.000	0.691 ± 0.008	0.291 ± 0.005
Default One hot	0.902 ± 0.000	0.681 ± 0.001	0.690 ± 0.009	0.292 ± 0.007
Default ProtBERT	0.905 ± 0.000	0.696 ± 0.001	0.697 ± 0.008	0.298 ± 0.006
